# Association of inherited thrombophilia mutations and their combinations among palestinian women with unexplained recurrent miscarriage

**DOI:** 10.1186/s12959-024-00587-7

**Published:** 2024-02-13

**Authors:** Ayman A. Najjar, Imam Hassouna, Mahmoud A. Srour, Hany M. Ibrahim, Randa Y. Assi, Heba M. Abd El Latif

**Affiliations:** 1 Physiology Unit, Zoology Department, Faculty of Science, Menoufia University, Shibin El Kom, Egypt; 2 Department of Biology and Biochemistry, Faculty of Science, Birzeit University, Birzeit, Palestine; 3 Clinical Laboratory Science Program, Faculty of Pharmacy, Nursing and Health Professions, Birzeit University, Birzeit, Palestine; 4 Department of Obstetrics & Gynecology, Faculty of Medicine, Al-Quds University, Jerusalem, Palestine

**Keywords:** Inherited thrombophilia mutations, Recurrent miscarriage, Prevalence, Women, Palestine

## Abstract

**Background:**

Inherited thrombophilia (IT) has a complex pathophysiology and is associated with recurrent miscarriage (RM) by causing placental insufficiency and inhibiting fetal development. However, thrombophilia screening in unexplained RM cases is still questionable. This study aimed to investigate the association between the common eight IT mutations and their combinations among Palestinian women with unexplained RM.

**Methods:**

This is an unmatched case-control study with 200 women (100 unexplained RM cases, 100 controls). Eight common IT mutations namely Factor V Leiden (FVL), prothrombin gene (FII) G202120A, Methylenetetrahydrofolate Reductase (MTHFR) gene (C677T and A1298C), B-fibrinogen gene − 455G > A, FV HR2 A4070G, Plasminogen activator inhibitor 1 (PAI1) 5G/4G and Factor XIIIA (FXIIIA) V34L; were analyzed. The first five mutations were analyzed by Restriction Fragment Length Polymorphism PCR and the other three mutations were analyzed using Amplification Refractory Mutation System PCR.

**Results:**

The prevalence of the eight IT mutations among the control group was in the order PAI1 5G/4G (69%), MTHFR C677T (53%) and A1298C (47%), BFG − 455G > A (35%), FVL and FV HR2 (each 18%), FXIIIA V34L (16%) and FII G20210A (3%). Patients had a higher percentage of MTHFR A1298C (heterozygotes and mutant homozygote) compared to controls (*p* = 0.016). Frequencies of mutant alleles MTHFR A1298C (*p* < 0.001) and FXIIIA V34L (*p* = 0.009) were higher among patients compared to controls. No significant differences were observed for all other mutations or mutant alleles. Most patients (75%) and controls (75%) have 2–4 mutant alleles out of 8 mutant alleles studied, while 1% of patients and 2% of controls have zero mutant alleles. None of the combinations of the most often studied mutations (FVL, FII G20210A, MTHFR C1677T, and MTHFR A1298C) showed a significant difference between patients and controls.

**Conclusions:**

There was a significant association between unexplained RM and the mutant alleles of MTHFR A1298C and FXIIIA V34L. No significant association was observed between unexplained RM and the combination of both mutant alleles for the mutations studied. This study is the first Palestinian report that evaluates eight inherited thrombophilia mutations and their alleles’ combinations in unexplained RM cases.

## Introduction

Recurrent miscarriage (RM), unlike sporadic pregnancy loss, necessitates additional attention, monitoring, and follow-up in subsequent pregnancies to be successful [1, 2]. RM is a disappointing experience that negatively impacts couples’ psychological status and worsens their social discomfort [3]. RM is classified into primary and secondary: primary RM is the repeated miscarriage without viable previous babies, but secondary RM is the repeated pregnancy loss with a live birth at some time [3]. Secondary RM has a better prognosis than primary RM, in fact, primary RM is a poorly understood condition, which affects 1–3% up to 5% of couples trying to have children worldwide [3–14]. RM is defined by the American Society of Reproductive Medicine (ASRM) and the European Society for Human Reproductive and Embryology (ESHRE) as the loss of two or more consecutive pregnancies from conception to 24 weeks of gestation [1, 4–9, 11, 13–17]. While the World Health Organization (WHO) and the Royal College of Obstetricians and Gynecologists (RCOG) define RM as three or more miscarriages verified by ultrasonography or histology up to 24 weeks of gestation [2, 4, 5, 15, 16, 18–20]. As a result, several measurements and recommendations were developed and implemented to reach standardization for appropriate inquiry and treatment approaches [1, 15].

In normal pregnancy, the hemostatic system is modified to a hypocoagulable condition to preserve oxygen and nutrition transmission via the placenta for fetal survival, then changed to a hypercoagulable state to prevent excessive bleeding following birth [2, 6, 10, 21–23]. Because of the interaction of various risk factors that play a role in RM such as maternal age, lifestyle behaviors (stress, smoking, and excessive alcohol consumption), history of miscarriage, antiphospholipid syndrome, uterine malformation, endometritis, endocrinological, abnormal parental karyotypes, obesity, genetic factors, and thrombophilia with other unknown factors, RM is considered a multifactorial condition [1–5, 7–9, 11, 12, 14, 15, 17, 19, 24, 25]. Inherited thrombophilia (IT) is a hypercoagulopathy state that increases the risk of thrombosis. The combination of physiological alterations in hemostatic systems with IT in pregnancy has been reported to increase the risk of pregnancy complications in 40–50% [6, 24] or even 80% of cases [12].

RM is a multi-etiological syndrome with more than 50% of cases remaining unexplained, with the underlying processes remaining unknown, limiting the diagnosis and treatment protocols, and creating a challenge to patients and doctors [1, 3, 4, 6–8, 12, 14–16, 18, 20, 24, 26]. RM demonstrates significant causative variability in known and unknown etiology groups [15, 16]. Despite substantial research on the subject, studies are still limited, suffer from population selection bias, use diverse diagnostic criteria, and yield conflicting conclusions [4].

Inherited or acquired thrombophilia causes irregular blood coagulation and an increased risk of venous thromboembolism (VTE). IT is caused by mutations that affect gene function in the anticoagulant mechanism, causing the hemostatic system to become thrombotic [11, 21]. Thrombophilia affects about 5% of the general population [3]. Prothrombin gene (FII) mutation (G20210A) and factor V Leiden (FVL) were detected in around 50–70% of VTE cases diagnosed with inherited thrombophilia [21]. Pregnancy is associated with around 20% of thrombotic events; additionally, pregnancy raises the incidence of VTE by 5 times [12, 18, 21]. It is estimated that around 40–50% of VTE caused by IT occurs during pregnancy [3, 12, 21]. The association between thrombophilia and RM has been investigated by many studies with controversial conclusions [3, 11, 12]. Therefore, in cases of RM, a panel of laboratory tests belonging to hemostatic, and IT are usually recommended [3].

Clinically, most carriers of thrombophilia mutations will not show symptoms and will go untreated, but exposure to additional risk factors such as pregnancy may raise the likelihood of life-threatening complications to become clinically clear [12, 26]. IT panel includes FVL R506Q mutation, FII gene G20210A mutation, Methyltetrahydrofolate Reductase gene mutations (MTHFR C1677T; A1298C), B-fibrinogen gene − 455G > A, Plasminogen activator inhibitor 1 (PAI1) 5G/4G, Factor XIIIA (FXIIIA) V34L, and FV HR2 A4070G. FII G20210A, FVL and MTHFR C677T and A1298C are the most extensively studied thrombophilia mutations in RM, and most studies reported wide ranges of prevalence for each mutation among the respective study population with controversial findings [6, 10–16, 24, 26–30].

In the past 13 years, eight studies have been conducted regarding RM among Palestinian women in Palestine (West Bank and Gaza) [6–9, 25, 27, 31–33]. These reports examined one [9, 25, 27], two [7] three [6, 8, 33], or seven IT mutations [32] and reported controversial results.

About 2.4% of married Palestinian women experience primary infertility, and about 6.0% experience secondary infertility [34]. The incidence of miscarriage in the Palestinian community was estimated at 4–8% that is considered relatively high and necessitates more investigations to minimize miscarriage and increase the opportunity of normal pregnancy and delivery of live baby [27]. In Palestine, miscarriage cases have been attributed to several factors including thrombophilia (32.5%), followed by IVF pregnancies (8.8%), and twin pregnancies (8.6%) among other causes [34]. Government high-risk pregnancy clinics received referrals from 17.4% of pregnant Palestinian women in 2018 [34]. In the investigation of unexplained RM cases, analysis of IT has been a common approach. Therefore, the aim of our study was to investigate the association between the common eight IT mutations and their combinations among Palestinian women with unexplained RM.

## Materials and methods

### Study design and setting

Unmatched case-control study was designed with 200 women recruited from the West Bank region of Palestine (northern, central, and southern), that includes eleven governorates, between February 2020 to October 2021. The study sample included 100 cases with unexplained RM women and 100 controls. As of June 2019, the population of Palestine (West Bank) was 2,986,714 [34]. Females made up 1,463,489 (49%) of the entire population, and 715,646 (48.9%) of them are women of reproductive age (15–49 years old). 443,700 women were married and of reproductive-age [34]. Using the WHO criteria as a guide, a sample size calculation was expanded to 200 participants (100 cases and 100 controls), with 95% level of significance, 80% power, and a minimum OR of 2.0 [35]. In accordance with each governorate’s population, study participants were recruited from each of the eleventh West Bank’s governorates to achieve a statistically representative sample of the West Bank population.

Women (*n* = 100) with at least three unexplained RM before the 24th week of gestation were included in this study. Additionally, healthy women (*n* = 100) were selected as the control group from the same regions as the study group (patients). Controls had more than two live babies and had no previous history of miscarriage. All subjects supplied a written informed consent. The study protocol was re-evaluated and accepted by the local ethics committee of the Palestinian Medical Technology Association.

### Sampling procedure

All participants underwent a brief interview, were requested to provide written informed consent, and subsequently underwent evaluation by a gynecologist to ascertain their eligibility based on the inclusion criteria. Subsequently, an interview-based questionnaire was administered, and 5 mL of venous blood was collected using the vacutainer tube method and in accordance with the relevant standard operating procedures. The EDTA tubes were labeled with the participant’s full name and serial number. To prevent clumping and clot formation, the EDTA samples were gently mixed for 10 min using a dedicated mixer.

### Genotyping of inherited thrombophilia mutations

Genomic DNA was prepared from whole blood using the Genomic DNA mini-Kit (Geneaid, Taiwan), per the manufacturer’s instructions. Amplification Refractory Mutation System (ARMS) -PCR and Restriction Fragment Length Polymorphism (RFLP) -PCR were used for genotyping the IT mutations. Briefly, ARMS-PCRs was used to genotype FV HR2 [36] and PAI1 5G/4G [37] and RFLP–PCR was used to genotype FVL [38], MTHFR C677T and A1298C [39] and BFG − 455G > A [40] mutations as described earlier. FXIIIA V34L mutation was analyzed using newly designed ARMS primers that generate a 266-bp amplicon: 5’- ctg ccc aca gtg gag ctt cag *a*gc g -3’ for the wild type primer; 5’- ctg ccc aca gtg gag ctt cag *a*gc t-3’ for the mutant primer and 5’- cag aac tgg gac aag gct ctg ggt c-3’ for the reverse common primer. The wild type and mutant primers contain a mismatch at position − 4. The ARMS PCR reaction was performed using HotStart PCR mix (Bioneer, S.Korea) using 0.25 µM of each primer and 100–150 ng of genomic DNA in a 20-µL reaction. Thermal cycling was done using Biorad T100 machine as follows: 94 °C for 10 min, 30 cycles of 94 °C for 30 s, 62 °C for 45 s and 72 °C for 45 s; followed by a final extension step at 72 °C for 5 min. FII G20210A was genotyped using newly designed artificial RFLP PCR primers to generate a 264-bp amplicon: 5’- aca acc gct ggt atc aaa tgg gca tcg-3’ for the Forward primer and 5’- ctg ccc atg aat agc act ggg agc att gaa gc-3’ for the R primer. The nucleotide “G” at position (-3) in Reverse primer is replaced by “A” to generate a site for HindIII in the A allele (mutant). RFLP PCR was performed using HotStart PCR mix (Bioneer, S.Korea) using 0.25 µM of each primer and 100–150 ng of genomic DNA in a 20-µL reaction. Thermal cycling was performed in Biorad T100 machine as follows: 94 °C for 10 min, 15 cycles at 94 °C for 30 s, 55 °C for 45 s and 72 °C for 45 s; and 20 cycles at of 94 °C for 30 s, 56 °C for 45 s and 72 °C for 45 s; followed by a final extension step at 72 °C for 5 min. Positive and negative DNA controls were included in each experiment. Amplicons were digested with HindIII and restriction fragments were analyzed on 4.5% agarose gel and stained with ethidium bromide. The FII G20210A genotypes were based on size of restriction fragments as follows: 264 bp indicated a homozygote normal; 232 and 32 bp indicate a homozygote mutant, and 264, 232 and 32 bp indicate a heterozygote state.

### Data quality assurance

Personal protective equipment was consistently utilized. A proficient phlebotomist collected blood samples and adhered to the respective standard operating procedures. The quality of samples and reagents was maintained through implementation of the respective standard operating procedures.

### Data processing and analysis

The outcomes were recorded in an Excel spreadsheet and subsequently exported for analysis using the Statistical Package for Social Sciences program (SPSS) version 23. The ‘N-1’ Chi-square test was employed to compare the means of proportions. The correlation between the number of abortions and number of mutant alleles was analyzed using Pearson’s correlations. Odds ratios were calculated to measure the association between exposure (mutation) and outcome (abortion) for mutations that showed significant differences between cases and controls. Statistical significance was determined by P-values less than 0.05.

### Ethical approval

The study protocol received approval from the local ethics committee of the Palestinian Medical Technology Association (file #26/2020). Prior to enrollment, participants were duly informed of the study’s objectives and provided a written informed consent. To ensure confidentiality, all information was anonymized.

## Results

### Characteristics of the study population

A total of 200 female participants were enrolled in this study, comprising of 100 women who had unexplained RM with three or more consecutive miscarriages before 24 weeks of gestation, and 100 healthy women who had at least two children and no history of adverse pregnancy outcomes or miscarriages. The mean age of the patients and controls were 29.4 (range 20–42) and 31.9 (range 21–45) years, respectively. To ensure a representative sample of the study region (West Bank) and to eliminate any potential environmental factors that may impact the measured parameters, study subjects were recruited from various governorates in the West Bank region, Palestine. There were no significant differences between the patients and controls in terms of family history of abortion, family history of cardiovascular disease, consanguineous marriage, body mass index, diabetes mellitus, hypertension, smoking, and coffee consumption [41]. Most patients (75%) had experienced 3–4 miscarriages, while 9% had 7 or more. All participants (patients and controls) underwent laboratory testing for thyroid disorders, antiphospholipid syndrome, autoimmune diseases, platelet parameters, and other coagulation and fibrinolytic status. However, significant differences between the two groups were observed in Protein C and S, D-dimer, anticardiolipin (IgM, IgG), and antiphospholipid antibody (IgM) [41].

### Inherited thrombophilia mutations

**Fig. 1:**
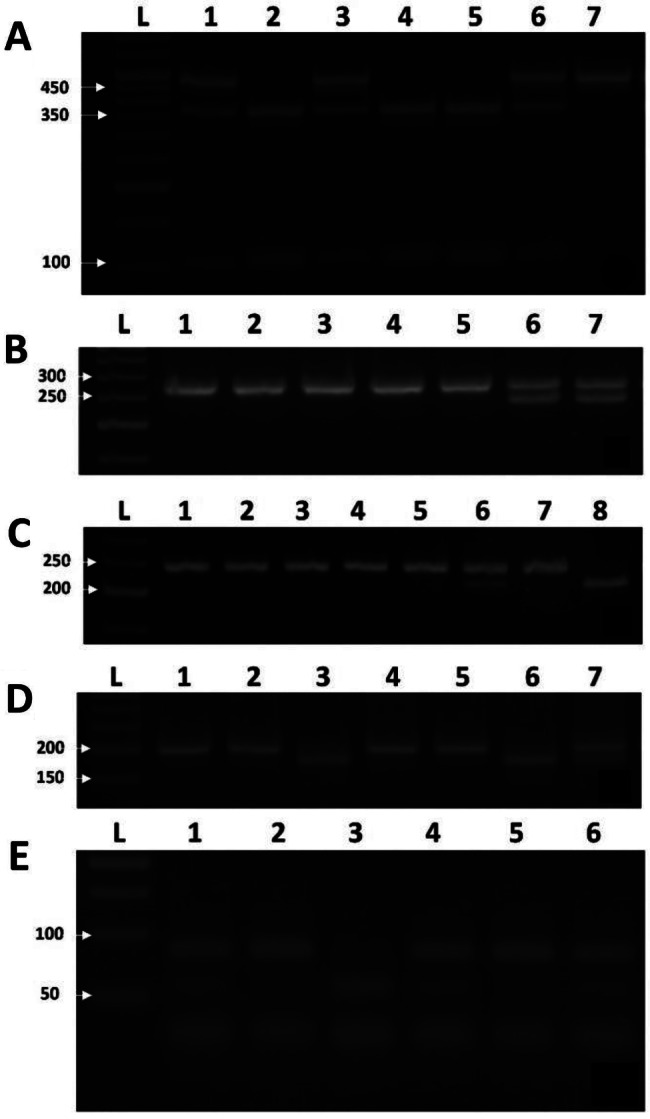
Representative agarose gels of RFLP PCR. (**A**) BFG-455G > A mutation: *HaeIII* digestion of PCR product (446 bp) yields 348 and 98 bp for the wild type allele or 446 bp for the mutant allele. Lane L: 50 bp DNA ladder; lanes 2, 4 and 5 are normal; lanes 1, 3 and 6 are heterozygote; and lane 7 is mutant homozygote. (**B**) FII G20210A mutation: *HindIIII* digestion of PCR products yields 264 bp for the wild type allele and 232 and 32 bp for the mutant allele. Lane L: 50 bp DNA ladder; lanes 1–5 are normal; lanes 6 and 7 are heterozygotes. (**C**) FVL mutation: *HindIII* digestion of PCR products yield 241 bp for the wild type allele and 209 and 32 bp for the mutant allele. Lane L: 50 bp DNA ladder; lanes 1–5 and 7 are normal; lane 6 is heterozygote; and lane 8 is mutant homozygote. (**D**) MTHFR C677T mutation: *HinfI* digestion of PCR products yields 198 bp for the wild type allele and 175 and 23 bp for the mutant allele. Lane L: 50 bp DNA ladder; lanes 1, 2, 4 and 5 are normal; lane 7 is heterozygotes; and lanes 3 and 6 are homozygote mutants. (**E**) MTHFR A1298C mutation: *MboII* digestion of PCR products yields 56, 31, 30, 28 and 18 bp for the wild type allele and 84, 31, 30 and 18 bp for the mutant allele. Lane L: 50 bp DNA ladder; lane 3 is normal; lanes 1, 4 and 6 are heterozygotes; and lanes 2 and 5 are homozygote mutants

**Fig. 2:**
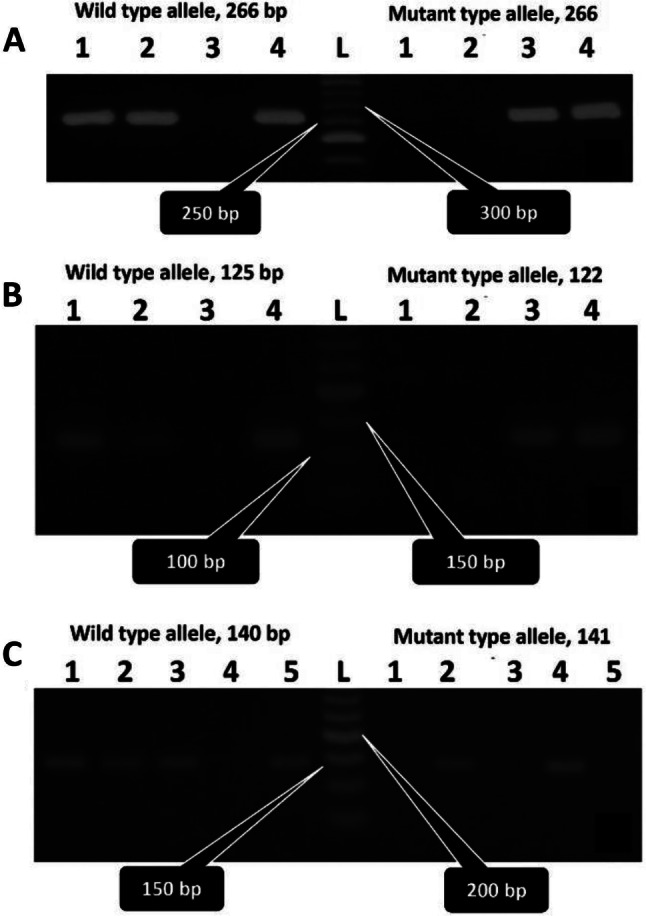
Representative agarose gels of ARMS PCR. (**A**) FXIIIA V34L mutation; lane L: 50-bp DNA ladder; lanes 1 and 2 are normal; lane 3 is mutant homozygote; and lane 4 is heterozygote. (**B**) FV HR2 mutation; lane L: 50-bp DNA ladder; lanes 1 and 2 are normal; lane 3 is mutant homozygote mutant; and lane 4 is heterozygote. (**C**) PAI1 5G/4G mutation; Lane L: 50-bp DNA ladder; lanes 1, 3 and 5 are normal; lane 2 is heterozygote; and lane 4 is mutant homozygote

The IT mutations were determined using either RFLP PCR or ARMS PCR and a representative agarose gel for each mutation is shown in Figs. 1 and 2, respectively.

To investigate the prevalence of IT mutations among healthy Palestinians, we assessed the prevalence of the main eight common IT mutations among the control group in this study as shown in Tables 1 and 5. PAI1 5G/4G mutation showed the highest prevalence with 69% (19% homozygous and 50% heterozygous), followed by MTHFR C677T mutation (53%) and the MTHFR A1298C (47%) (Table 1). The frequency of FVL heterozygotes was 18% and homozygote FVL was not detected among the control group. FII G20210A showed the lowest prevalence in this study.

Statistical analysis of the main eight thrombophilia mutations analyzed in this study, revealed that only the proportion of mutant MTHFR (A1298C) (heterozygotes and mutant homozygote) has a significant difference between patients and controls (*p* = 0.016), with a prevalence of 64% among patients (48% heterozygous and 16% homozygous) and 47% among controls (37% heterozygous and 10% homozygous) (Table 1). MTHFR C667T and FXIIIA V34L homozygous mutations showed obvious differences between patient and control groups (*p* = 0.055) but it was not statistically significant (Table 1). The other tested thrombophilia mutations displayed no statistically significant differences between the two study groups. The proportions of FXIIIA V34L (*p* = 0.059) and BFG − 455G > A mutations (*p* = 0.150) were higher among patients (27% and 45%, respectively) compared to controls (16% and 35%, respectively) but it was not statistically significant.

**Table 1:** Prevalence of hereditary thrombophilia mutations among unexplained RM patients and controls

Mutation	Patients	Controls	P value	Patients	Controls	P value	Patients	Controls	P value
	Mutant %			Hetero %			Homo Mutant %		
FVL R506Q	20	18	0.719	19	18	0.856	1	0	0.317
FII G20210A	1	3	0.314	1	3	0.314	0	0	1.0
MTHFR C677T	51	53	0.778	43	36	0.313	8	17	0.055
MTHFR A1298C	**64**	**47**	**0.016**	48	37	0.117	16	10	0.208
BFG − 455G > A	45	35	0.150	36	26	0.127	9	9	1.0
PAI1 5G/4G	63	69	0.372	48	50	0.778	15	19	0.453
FXIIIA V34L	27	16	0.059	21	15	0.271	6	1	0.055
FV HR2 H1299R	15	18	0.569	13	17	0.429	2	1	0.562

Data are expressed as Mutant %: Mutant includes both heterozygotes and mutant homozygotes; Hetero %: Heterozygous %; Homo mutant %: Homozygous mutant. Data was analysis by the N-1 chi-squared test and a p value < 0.05 was considered significant. The p-value in bold font was used to stress the significant values or values less than 0.05.

Analysis of the mutant allele frequencies among the study subjects showed a statistically significant difference in the frequencies of mutant alleles of MTHFR A1298C (*p* < 0.001) and FXIIIA V34L (*p* = 0.009) between patients and controls (Table 2). Furthermore, analysis of odds ratios revealed that patients with MTHFR A1298C mutant allele have a 2-fold risk of RM (odds ratio 2.005; 95% CI 1.138–3.533), while a significant risk of RM was not observed with the FXIIIA V34L mutant allele (odds ratio 1.942; 95% CI 0.971–3.884). Remarkably, the allele frequency of BFG − 455G > A mutant allele was higher in patients compared to controls, while the frequencies PAI1 5G/4G and MTHFR C677T mutant alleles were lower in patients compared to controls, however, these differences were not significant (Table 2).

**Table 2:** Frequency of mutant alleles among study subjects

Mutation	Mutant allele frequency (%)		P-value
	Patients	Controls	
FVL R506Q	21	18	0.593
FII G20210A	1	3	0.314
MTHFR C677T	59	70	0.104
MTHFR A1298C	80	57	**< 0.001**
BFG − 455G > A	54	44	0.158
PAI1 5G/4G	78	88	0.060
FXIIIA V34L	33	17	**0.009**
FV HR2 H1299R	17	19	0.714

Data are expressed as alleles where normal genotype was represented by zero alleles and hetro and homo mutants were represented by one and two alleles, respectively. Total no. of alleles was calculated by multiplying the number of alleles by the number of patients or controls. Data was analysis by the N-1 chi-square test and a p value < 0.05 was considered significant. The p-value in bold font was used to stress the significant values or values less than 0.05.

Examination of differences between patients and controls, based on the number of mutant alleles among the eight IT mutations investigated in this study, revealed no statistically significant differences (Table 3). The highest proportion of patients (33%, 25%) and controls (29%, 24%) have 3 and 4 mutant alleles, respectively. However, the proportion of patients and controls with zero mutant alleles was 1% and 2%, respectively.

**Table 3:** Frequency and proportion of hereditary thrombophilia alleles among patient and control groups

No. of alleles mutants	Patients, %	Controls, %	P-value
0 mutant alleles	1	2	0.819
1 allele mutant	9	9	1.000
2 alleles mutant	17	22	0.761
3 alleles mutant	33	29	0.845
4 alleles mutant	25	24	0.957
5 alleles mutant	8	10	0.856
6 alleles mutant	4	3	0.889
7 alleles mutant	3	1	0.610

Total Alleles were calculated by multiplying the number of alleles by the number of patients or controls. Data was analysis by the N-1 chi-squared test and a p value < 0.05 was considered significant

Analysis of the combination of the most frequently studied IT mutations (FVL, FII G20210A, MTHFR C1677T, and MTHFR A1298C) among patients and controls showed no significant difference (Table 4). In addition, upon further analysis of potential combination scenarios of all IT mutations studied, no statistically significant differences between patients and controls were (data not shown). However, the highest percentage of combination scenarios observed was the “MTHFR C677T Hetero + MTHFR A1298C Hetero + normal FVL + normal FII G20210A” and it was observed in 16% of patients and 12% of controls.

**Table 4:** Proportions of the combinations of mutant alleles belonging to FVL, FII G20210A, MTHFR C1677T, and MTHFR A1298C, in patient and control groups

Number of alleles	Patients, %	Controls, %	P-value
1 mutant allele	34	33	0.881
2 mutant alleles	48	45	0.671
3 mutant alleles	9	7	0.603
4 mutant alleles	1	0	0.317

Total Alleles were calculated by multiplying the number of alleles by the number of patients or controls. Data was analysis by the ‘N-1’ chi-squared test and a p value < 0.05 was considered significant

The correlation between the number of abortions and number of mutant alleles was analyzed using Pearson correlation among the patient group. No significant correlation was observed between the number of abortions and total number of mutant alleles (for all eight IT mutations analyzed), *R*(98) = − 0.069, *p* = 0.496; and number of mutant alleles of the main four mutations (FVL, FII G20210A, MTHFR C677T and A1298C), *R*(98) = − 0.078, *p* = 0.439.

## Discussion

The impact of lifestyle factors on sporadic miscarriage is well-established, however, their contribution to RM remains unclear. This study aimed to investigate this point by examining a group of patients (aged 20–42 years) and controls (aged 21–45 years) who were of similar ethnic and social backgrounds and residing in different districts of the West Bank. The sample was selected using statistical methods to ensure it was representative of the West Bank population. Anthropometric data and various lifestyle factors were analyzed for both groups, consisting of 100 patients and 100 controls. The study found no significant differences between patients and controls in terms of family history of abortion, consanguineous marriage, diabetes mellitus, hypertension, family history of cardiovascular disease, anticoagulant use, coffee consumption, and folate supplementation [41].

IT is a coagulation disorder related to hypercoagulopathy state and thrombotic events. It has a complex pathophysiology status and is associated with RM by causing placental insufficiency and inhibiting fetal development. Thrombophilia screening is still questionable, so laboratories perform different panels of tests under the name of thrombophilia in unexplained RM cases [14].

RM is a global health problem that affects all communities worldwide and requires sharing efforts for real effective solutions, but because of the different genetic backgrounds between people of the world, the contribution of IT mutations to RM is expected to differ among ethnic groups. Therefore, it would be helpful to compare the prevalence of IT mutations locally, regionally, and globally to identify similarities and differences between Palestine and other countries. This may facilitate a better understanding and management of unexplained RM.

In our study, we investigated the prevalence of the common eight IT mutations among healthy controls. The most frequent mutations were PAI1 5G/4G (69%), MTHFR C677T (53%), and MTHFR A1298C (47%), while FII G20210A displayed the lowest frequency (3%) (Tables 1 and 5). Our findings exhibit that the most common IT mutations analyzed (FVL, FII G20210A, MTHFR C677T, and MTHFR A1298C) were consistent with the prevalence rates reported by earlier studies from Palestine except for FII G20210A mutation which showed a lower prevalence in our study (3%) compared to earlier Palestinian studies (Table 5) [6, 32, 33]. The prevalence of PAI1 5G/4G and FV HR2 mutations among the Palestinian population was not reported previously. Also, the prevalence of the main four IT (FVL, FII G20210A, MTHFR C677T and A1298C) among Palestinians was within the range observed in neighboring Arab countries, namely Lebanon [24], Jordan [42], Syria [43] and Egypt [44]. These neighboring Arab countries and Palestine have similar genetic backgrounds (Table 5). However, the prevalence of the three main inherited thrombophilia mutation (FVL, FII G20210A and MTHFR C677T) among healthy Palestinians and healthy populations in other regions of the world (Europe and America) [45] revealed that FVL and MTHFR C677T prevalence ranked Palestine among countries with high prevalence of these mutations, but FII G20210A prevalence was consistent with other regions as shown in Table 5 [45]. Variations in the prevalence of IT mutations among diverse populations can be attributed to the distinct genetic profiles of the populations under investigation. Thus, the determination of the prevalence of these mutations in each population should aid in the development of protocols for thrombophilia screening in the respective population.

**Table 5:** Comparison of the prevalence of the most common inherited thrombophilia mutations among healthy Palestinian women (healthy controls) observed in this study to that reported by previous Palestinian studies, neighboring Arab countries, and other regions of the world

		Palestinian studies			Neighboring Arab countries				Regions of the world	
Mutation	This study	Ref. [6]	Ref. [32]	Ref. [33]	Lebanon Ref. [24]	Jordan Ref. [42]	Syria Ref. [43]	Egypt Ref. [44]	Europe Ref. [45]	America Ref. [45]
FVL R506Q	18	18.2	23.6	20.1	9.0	30	11.5	58.1	5–9	3-5.2
FII G20210A	3	4.2	5.9	9.3	2.0	8.3	2.5	4.7	2–6	NA
MTHFR C677T	53	NA	50.9	13.8	63.0	28.3	37.7	58.2	10–16	NA
MTHFR A1298C	47	NA	49.0	NA	NA	58.2	38.8	NA	NA	NA

Data are expressed as Mutant %: Mutant includes both heterozygotes and mutant homozygotes; Hetero %: Heterozygous %; Homo mutant %: Homozygous mutant. NA: not available

In addition, the study investigated the association between the main eight inherited thrombophilia mutations and unexplained RM among Palestinian women from the West Bank region of Palestine compared to control women with normal deliveries. Our results indicated a significant association between MTHFR A1298C and unexplained RM in the Palestinian population (p 0.016). In contrary, the other inherited thrombophilia mutations analyzed in this study (FVL, FII G20210A, and MTHFR C677T, BFG − 455G > A, PAI1 5G/4G, FXIIIA V34L, FV HR2) displayed no significant association with unexplained RM. Thrombophilia in general (inherited and acquired) is diagnosed in about 32.5% of miscarriage cases in Palestine [34]. So far, the six Palestinian previous studies that examined the prevalence of IT and its association with RM showed conflicting results for their association with RM, but they showed consistent findings concerning the prevalence of inherited thrombophilia mutations among healthy women [6–8, 27, 32, 33]. Thus, this study confirmed local previous reports on MTHFR A1298C that reported a significant association of this mutation and RM [32], as well as no significant association with MTHFR C677T [6], FVL [8, 27], FV HR2 [32], FII G20210A [6, 8, 32] and BFG − 455G > A [7, 32]. Our results concerning FVL, FII G20210A and MTHFR C677T were also in contrary to reports from other previous studies [3, 10, 11, 15, 17, 26].

For further investigation of the association between unexplained RM and inherited thrombophilia mutations, we analyzed the frequency of mutant alleles for the common eight mutations. Our findings revealed that the frequencies of mutant alleles of MTHFR A1298C (*p* < 0.001) and FXIIIA V34L (p 0.009) were significantly higher in patients compared to controls (Table 2). Additionally, the mutant allele MTHFR A1298C was associated with a 2-fold increased risk (odds ratio 2.005; 95% CI 1.138–3.533) of RM, while such an effect was not observed with FXIIIA V34l (odds ratio 1.942; 95% CI 0.971–3.884). Most subjects in the patient (75%) and control groups (75%) have 2–4 mutant alleles out of the eight mutant alleles analyzed here; and only 1% of patients and 2% of controls have zero mutant alleles out of the eight alleles studied Table 3). In addition, we have analyzed the combination of the most frequent mutant alleles of FVL, FII G20210A, MTHFR C677T and A1298C (Table 4), and no significant association was observed either between the number of mutations nor the number of their alleles with unexplained RM. The most frequent combination of alleles was 1–2 alleles and it was observed in 82% of patients and 78% of controls (Table 4).

A recent bibliometric analysis study by Deng et al. [23] analyzed 725 articles performed in the last 30 years and published by 3205 authors from 1139 organizations and 68 countries; and concluded that there is a clear causal relationship between thrombophilia and RM and tests evaluating thrombophilia should always be considered. In addition, the authors advise investigation of the unknown thrombophilia status of controls [23]. A systematic review of the literature that included only case-control studies, 16 articles were selected for the FVL and 7 for the FII G20210A analysis, and the authors concluded that women with either one of these mutations have a 2-fold risk of RM and recommended testing for these mutations in RM cases [26]. On the other side, a retrospective cohort study of 1155 women with RM in the UK reported that the prevalence of inherited thrombophilia in women with RM and the general population are similar and [19].

Controversial findings are commonly encountered with association studies and especially if conducted on different populations and ethnic groups [7]. These variations can be attributed to different genetic backgrounds of the different populations or ethnic groups studied, as well as environmental factors and genetic modifiers, especially with a multifactorial condition like RM. Therefore, the findings of inherited thrombophilia mutations in patients should be interpreted within the context of the clinical manifestation of each patient. Also, most studies that tackled the role of inherited thrombophilia in RM have focused on a small group of mutations [3, 10, 11, 17, 24, 26, 29], namely FVL, FII G20210A, MTHFR C677T and A1298C, while studies that investigated a larger panel of thrombogenic mutations are rare [15]. Consequently, the later four main mutations have been extensively studied and several studies have reported risk values for them [3, 10, 15, 17, 24, 26, 29], albeit with wide variations, while such a risk value is not available for other mutations. In clinical practice, thrombophilia screening of RM cases is still used, and the interpretation of the results is somehow arbitrary due to the lack of consensus risk values for the mutations analyzed or a consensus list of mutations that should be tested. Although, there are probably differences in the clinical relevance of the different types of thrombophilia as well as single or multiple IT mutations [23] and this may influence their interpretation. Additionally, most studies concerning IT as a hypercoagulable state are based on scientific facts and theories. While most gynecologists tend to manage RM cases with aspirin and heparin whether inherited thrombophilia mutations are detected or not [46–48]. Many studies do not support the use of anticoagulants or intravenous immunoglobulin in women with unexplained RM either with or without IT [46–54]. In exploring the possible causes of RM, it is interesting to note that several studies advise psychological supportive care alone for RM cases [46, 55].

A limitation of this study is the inability to analyze markers of hypercoagulopathy at different time points of pregnancy and link it to the findings of thrombophilia mutations, because most patients were not pregnant at the time of sample collection. Another limitation is that the low number of patients and controls that have FII G20210A mutation and thus the statistical analysis of this mutation is limited.

## Conclusion

The prevalence of six thrombophilia mutations analyzed in this study confirms earlier reports from Palestine and the prevalence of PAI1 5G/4G and FV HR2 mutations was reported for the first time in Palestine. Analysis of the association of the genotype or mutant alleles of eight inherited thrombophilia mutations with unexplained RM showed significantly higher frequencies of mutant alleles MTHFR A1298C and FXIIIA V34L in patients compared to controls. Also the MTHFR A1298C allele was associated with a 2-fold increased risk of RM, but no such effect was observed with FXIIIA V34l allele. No significant association between unexplained RM and the combination of either mutant alleles for the eight mutations studied or the four most common mutations (FVL, FII G20210A, MTHFR C677T, and A1298C) per patient was observed. Most unexplained RM cases (75%) and controls (75%) have 2–4 mutant alleles out of 8 mutant alleles studied, while only 1% of unexplained RM and 2% of controls have zero mutant alleles. This study is the first Palestinian report that evaluates eight IT mutations and their alleles’ combinations in statistically represented unexplained RM cases and controls.

The results of inherited thrombophilia mutations should be interpreted within the context of the findings of other laboratory tests and the clinical presentation of each unexplained RM case. Grading the risk of each IT mutation and the risk of their combinations would be more powerful to study the link of IT mutations with unexplained RM and may provide evidence for the most relevant set of mutations that should be analyzed in unexplained RM cases. Further studies are needed to explore other genetic modifiers that influence the effect of thrombophilia mutations and may explain the controversy observed concerning this point.

## Data Availability

The datasets analyzed during the current study are available from the corresponding author on reasonable request and institutional authorization.

## Abbreviations

RM: recurrent miscarriage
IT: inherited thrombophilia
FVL: Factor V Leiden
FV HR2: factor V HR2
MTHFR: Methylenetetrahydrofolate Reductase
FII: prothrombin gene or factor II
BFG: B-fibrinogen
PAI1: Plasminogen activator inhibitor 1
FXIIIA: factor XIIIA
RFLP: Restriction Fragment Length Polymorphism
PCR: polymerase chain reaction
ARMS: Amplification Refractory Mutation System
VTE: venous thromboembolism
WHO: World Health Organization
